# The Use of Ascorbic Acid in Adjunctive Treatment for Schizophrenia—Current State of Knowledge

**DOI:** 10.3390/life14070828

**Published:** 2024-06-28

**Authors:** Patrycja Piłat, Kamil Nikel, Joanna Smolarczyk, Magdalena Piegza

**Affiliations:** 1 Students Scientific Association, Department of Psychiatry, Faculty of Medical Sciences in Zabrze, Medical University of Silesia in Katowice, 40-055 Katowice, Poland; s79826@365.sum.edu.pl; 2 Department of Psychoprophylaxis, Medical University of Silesia in Katowice, 40-055 Katowice, Poland; joanna.smolarczyk@sum.edu.pl; 3 Department of Psychiatry, Faculty of Medical Sciences in Zabrze, Medical University of Silesia in Katowice, 40-055 Katowice, Poland; mpiegza@sum.edu.pl

**Keywords:** schizophrenia, ascorbic acid, vitamin C, clozapine

## Abstract

Schizophrenia is a mental illness characterized by disturbances in the perception of reality, thinking, emotions, and social functioning. This significantly impacts the quality of life of patients and leads to long-term disability. Despite advances in understanding its pathogenesis and treatment, schizophrenia remains a clinical challenge, especially due to the diversity of its symptoms and the complexity of its mechanisms. Schizophrenia is associated with abnormal functioning of the dopaminergic system, disturbances in glutamatergic neurotransmission, and oxidative stress in the brain. In recent years, there has been increasing interest in optimizing the treatment of mental disorders. The potential use of ascorbic acid, or vitamin C, in the therapy of schizophrenia could bring substantial benefits to patients. Ascorbic acid exhibits antioxidant and neuroprotective properties, suggesting its potential efficacy in reducing brain oxidative stress and improving neurotransmission. Additionally, there have been reports of its positive effects on psychotic symptoms and its potential in reducing the side effects of antipsychotic drugs. In this review article, we present the current state of knowledge on the potential use of ascorbic acid in the treatment of schizophrenia as an adjunct to standard pharmacological therapy. We analyze existing clinical studies and the mechanisms of action of vitamin C, suggesting its promising role as an adjunctive therapy in the treatment of schizophrenia. These insights, though not yet widely disseminated, may be significant for the further development of therapeutic strategies for this mental illness.

## 1. Introduction

Schizophrenia is one of the top ten causes of long-term disability worldwide. Its diverse symptoms include psychotic symptoms, apathy, isolation, and cognitive function disorders, significantly affecting the ability to function in society, work, and the need for self-care. Schizophrenia usually appears between the ages of 16 and 30, and its burdensome symptoms often accompany the patient throughout their life [1]. It affects approximately 24 million people, meaning 1 in 300 individuals (0.32%) globally suffer from this condition. This rate is 1 in 222 people (0.45%) among adults [2]. The dopamine hypothesis of schizophrenia suggests that dysfunctions in dopaminergic transmission play a key role in the development of symptoms of this disorder. It is believed that excessive dopaminergic activity in the mesolimbic system is the main cause of positive symptoms of schizophrenia, such as hallucinations and delusions, and antipsychotic drugs blocking dopamine receptors may reduce their intensity. At the same time, a dopamine deficiency in the prefrontal cortex is associated with negative and cognitive symptoms, such as apathy or difficulties with concentration. This hypothesis is supported by evidence from studies on the effects of amphetamines and brain imaging, although it is considered incomplete. According to the widely known dopamine hypothesis of schizophrenia symptom development, an excess of dopamine in the mesolimbic system is the primary cause of positive symptoms, whose intensity is reduced by antipsychotic drugs such as first-generation (typical) antipsychotics haloperidol, chlorpromazine, and fluphenazine and second-generation (atypical) antipsychotics including risperidone, olanzapine, quetiapine, aripiprazole, and clozapine that block dopamine receptors. Although these drugs are effective in alleviating many symptoms of schizophrenia, they are less effective in combating negative symptoms. Side effects of typical antipsychotics commonly cause extrapyramidal symptoms (EPSs), including tremors and tardive dyskinesia, and may also lead to neuroleptic malignant syndrome (NMS), which is potentially life-threatening. They can also cause sedation and anticholinergic effects like dry mouth and constipation. Atypical antipsychotics are associated with significant weight gain and metabolic syndrome, increasing the risk of diabetes and high cholesterol. While they have a lower risk of EPS compared with typical antipsychotics, they can still cause these symptoms to some extent. Clozapine, in particular, requires regular blood monitoring due to the risk of agranulocytosis, a severe drop in white blood cells [3]. Despite significant progress in the pharmacotherapy of schizophrenia, about 30% of patients respond inadequately to antipsychotic drugs and a high mortality rate is still observed among individuals with this disorder. The primary goal of schizophrenia treatment is to control the acute phase of the illness and prevent relapses. Antipsychotic drugs vary in efficacy and tolerance. Classical antipsychotic drugs, also known as first-generation, have a long history of use and act mainly through blocking dopamine receptors. On the other hand, atypical antipsychotic drugs, also known as second-generation, interact with various receptors, including serotonin receptors. As a result, they have the potential to alleviate both positive and negative symptoms of schizophrenia. However, both classical and atypical antipsychotic drugs can cause side effects such as metabolic disorders or motor system symptoms. Therefore, selecting the appropriate antipsychotic drug and monitoring the patient are crucial for effective therapy. Second-generation antipsychotics, such as aripiprazole, lurasidone, and paliperidone, are effective and safe in treating early-onset schizophrenia. Clozapine, a D_2_-5HT_2_ antagonist receptor, was the first antipsychotic drug to show efficacy in treatment-resistant patients and was associated with the relatively lowest risk of patient death [4]. Antagonizing 5-HT_2A_ receptors with clozapine can help reduce aggression, impulsivity, and negative psychosis symptoms. Clozapine also acts on dopaminergic receptors by antagonizing them, particularly D2, which reduces excessive dopaminergic activity associated with psychosis symptoms such as hallucinations and delusions. However, while clozapine’s antagonism of histamine H1 receptors can result in sedation and drowsiness in patients, its antagonism of muscarinic receptors can lead to side effects such as dry mouth, visual disturbances, constipation, and memory impairment. It is worth noting that these adverse effects tend to occur less frequently than with older-generation antipsychotics [5]. Available data indicate that clozapine is the treatment of choice for schizophrenia patients who exhibit violent behaviors or are at high risk of suicide. However, it is an antipsychotic drug with a high side-effect profile and a significantly high risk of complications, so its use must be well considered and in line with current treatment guidelines [4]. Given the many dilemmas in choosing the appropriate pharmacological therapy, a proper assessment of the dominant symptoms of psychosis and a comprehensive view of the patient’s functioning, including their treatment history and socioeconomic conditions are essential. Studies continue to emphasize the adverse impact of negative symptoms on the course of the disease, including functioning and fulfilling social roles [6]. Commonly used drugs help control symptoms, but their use is still associated with serious side effects such as weight gain, extrapyramidal symptoms, lipid metabolism disorders, endocrine problems, and sexual dysfunction. Olanzapine and clozapine are well known for causing weight gain in patients. Haloperidol is notorious for inducing EPS, including tremors and rigidity. Risperidone can also lead to EPS, particularly at higher doses. Both clozapine and olanzapine have been linked to disturbances in lipid metabolism, contributing to dyslipidemia. Olanzapine is known to disrupt endocrine function, leading to issues such as insulin resistance and hyperglycemia. Quetiapine has also been implicated in causing endocrine disturbances, including alterations in glucose metabolism. Risperidone is associated with sexual dysfunction, including decreased libido and erectile dysfunction. Aripiprazole, while less commonly than other antipsychotics, may also contribute to sexual dysfunction in some patients. In the search for new therapeutic solutions focusing on developing new neuroleptics, ascorbic acid [AA], or vitamin C, is currently one of the substances being studied as an adjunctive treatment in schizophrenia, showing both promising results and some reservations. AA performs antioxidant functions and participates in many metabolic processes in the human body. Its role includes involvement in collagen synthesis, iron absorption, supporting immune system function, and its antioxidant action, which protects cells from damage caused by free radicals. AA can affect the body’s ability to metabolize drugs through its action as an antioxidant [5]. AA influences glutamatergic transmission, which is crucial for proper brain function, including learning and memory processes. Glutamate is the main excitatory neurotransmitter in the central nervous system. Regulating its levels is important because excessive activation of glutamatergic receptors can lead to excitotoxicity and neuronal damage. AA modulates the action of the glutamate transporter in neurons and astrocytes, increasing the activity of the GLT-1 (EAAT2) transporter, which facilitates the uptake of glutamate from the synaptic space, reducing the risk of excitotoxicity. As a potent antioxidant, ascorbic acid protects nerve cells from oxidative stress, which is often associated with excessive activation of NMDA glutamatergic receptors. Reducing oxidative stress protects neurons from damage and apoptosis. AA can directly affect NMDA receptors, which are among the most important glutamatergic receptors. Modulation of the activity of these receptors by ascorbic acid contributes to the regulation of synaptic plasticity and neuroprotection. Additionally, AA influences the metabolism of other neurotransmitters, such as dopamine and serotonin, which may indirectly affect glutamatergic transmission. Through its influence on these neurotransmitter systems, AA may modulate overall synaptic activity in the brain. The role of AA as a neuromodulator of glutamatergic transmission has potential clinical significance in the therapy of neurodegenerative diseases such as Alzheimer’s disease and Parkinson’s disease, and in the treatment of disorders associated with excitotoxicity such as stroke. The neuroprotective properties of AA are also being investigated in the context of mental health, including the treatment of depression and schizophrenia [6,7]. The recommended intake level of vitamin C varies depending on age, sex, and physiological state. For an adult male over 19 years old, it is 90 mg/day, and for an adult female over 19 years old, it is 75 mg/day [8]. As part of a literature review conducted to explore the current knowledge on this topic, we discovered promising information regarding the potential impact of AA on the course of schizophrenia. 

## 2. Materials and Methods

**Figure 1:**
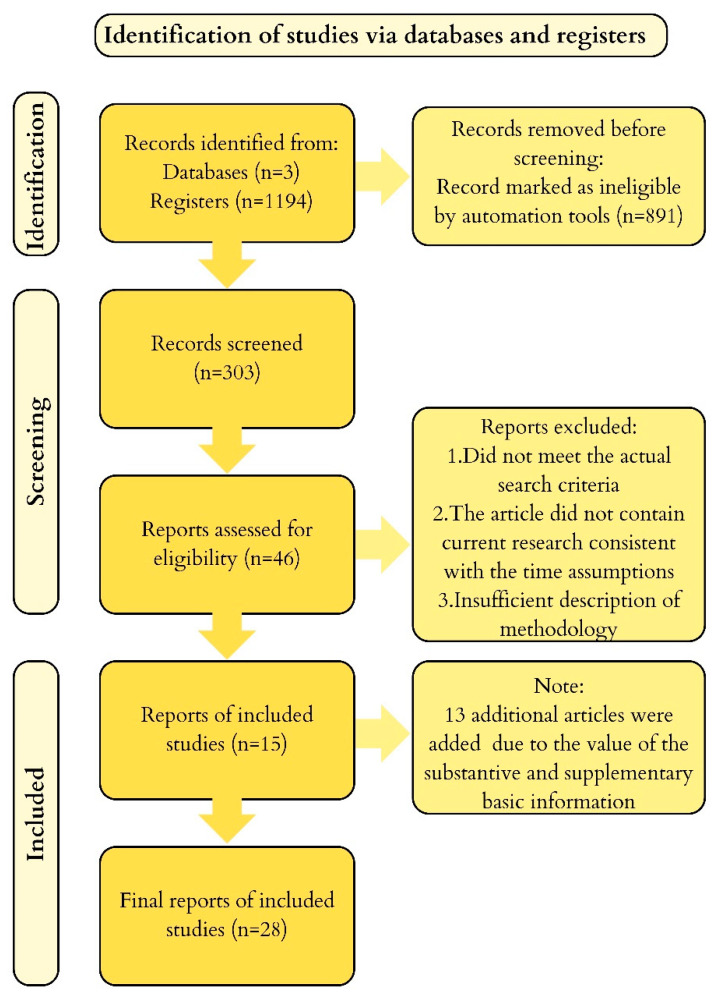
PRISMA diagram—selection of articles used in the review.

The work has the character of a systematic review. We searched Pubmed, Embase, and CINAHL (EBSCO). As of 2 April 2024, after entering the keywords “ascorbic acid, schizophrenia” into Pubmed and Embase, we obtained 128 and 470 records respectively. Similarly, after entering “ascorbic acid, clozapine” into Pubmed, Embase, and CINAHL, we obtained 26, 142, and 33 records respectively. We did not apply any time restrictions to the analysis of articles, and we only used full-text English-language manuscripts. In the selection process, we included clinical studies and randomized controlled trials involving individuals with schizophrenia who were administered ascorbic acid or placebo as an adjunct to standard antipsychotic treatment. We also included case reports. We did not consider studies that ambiguously determined the effect of vitamin C on schizophrenia (e.g., in the case of studies using a vitamin mixture without distinguishing the results of individual vitamins). In our review, we ultimately included 15 records of varying quality and sample size. The selected research papers contained statistically significant results and directly related to the topic of vitamin C supplementation in individuals with schizophrenia. The process of selecting articles used in the review is illustrated in the PRISMA diagram (Figure 1). The various doses of vitamin C and clozapine reported in the literature are summarized in Table 1.

The aim of the study was to critically evaluate the effects of using ascorbic acid to enhance neuroleptic treatment with clozapine in individuals with schizophrenia. Clozapine is often considered the first-line treatment for treatment-resistant schizophrenia due to its effectiveness in reducing both positive and negative symptoms, as well as preventing relapses of the disease. Additionally, clozapine is one of the most extensively studied antipsychotic medications, making it a significant reference point in research on new therapies or synergistic interactions with other substances.

## 3. Discussion

Vitamin C may exhibit certain potential benefits as an adjunctive treatment in schizophrenia, although its exact impact is not fully understood. Some studies suggest that vitamin C may have antioxidant, anti-inflammatory, and neuroprotective effects, which may be associated with symptomatic improvement in schizophrenia. It should be noted that limited research is reported in the available literature regarding the use of ascorbic acid combined with clozapine in the treatment of schizophrenia.

### 3.1. Vitamin C as an Antioxidant

There is evidence suggesting that individuals with schizophrenia may have elevated levels of oxidative stress, which may contribute to neuronal damage. Compounds associated with oxidative stress in schizophrenia include reactive oxygen species (ROS) such as superoxide radicals, hydrogen peroxide, and hydroxyl radicals, as well as reactive nitrogen species like nitric oxide. These compounds can induce oxidative damage to lipids, proteins, and nucleic acids, ultimately leading to neuronal dysfunction and cell death. As a potent antioxidant, ascorbic acid may help reduce this oxidative stress and protect nerve cells from damage [25]. In their study, P. Heiser et al. assessed the effects of using vitamin C to mitigate oxidative stress induced by antipsychotic medications. They utilized the highest available concentrations of each medication (haloperidol, clozapine, and olanzapine), combined with vitamin C (1 mM) to evaluate its impact. Additional incubation of rat blood with vitamin C showed a significant reduction in ROS production with haloperidol and clozapine. In the case of olanzapine, the difference did not reach statistical significance, but it also reduced the amount of ROS. These results suggest that antipsychotic medications may lead to increased ROS production, which may be associated with their toxic effects; however, this depends on the dose and the cell systems and organs studied. Ascorbic acid has demonstrated its significance as a cytoprotective factor against ROS [9]. It possesses the ability to inhibit the peroxidation of membrane phospholipids and function as a scavenger of free radicals. It plays a crucial role in suppressing superoxide radicals by blocking the auto-oxidation of catecholamines, thereby inhibiting the formation of potential toxic by-products such as 6-hydroxydopamine (6-OHDA), semiquinone, hydrogen peroxide, and hydroxyl radicals, which can contribute to the development of deficit symptoms. This may be one of the reasons for the improvement in Brief Psychiatric Rating Scale (BPRS) scores after vitamin C supplementation [10]. 

Superoxide dismutase (SOD) serves as a pivotal intracellular antioxidant enzyme, albeit present in small quantities extracellularly.In response to increased production of superoxide radicals and subsequent membrane damage caused by lipid peroxidation, there may be a compensatory increase in the concentration of extracellular SOD. In reaction to the increased production of superoxide radicals and subsequent membrane damage caused by lipid peroxidation, there could be a compensatory increase in SOD concentration in the extracellular environment. Lipid peroxidation may result in the formation of products of lipid peroxidation, such as malondialdehyde (MDA) and 4-hydroxynonenal (4-HNE), which are known markers of oxidative stress and cellular damage. However, the direct link between this phenomenon and the findings of aberrant free radical mechanisms in schizophrenia, as elucidated by Ganesh Dakhale et al., warrants further elucidation in the context of the reviewed literature [26]. A year after that report, Dakhale et al. published another study on schizophrenia. Patients with schizophrenia were found to have elevated levels of malondialdehyde (MDA) in serum and decreased levels of vitamin C in serum. After supplementation with vitamin C (500 mg/day) in combination with atypical antipsychotic drugs, significant reversal of these changes was observed compared with the group receiving placebo only with the atypical antipsychotic drugs. Additionally, the results of the changes assessed using the psychiatric rating scale after 8 weeks statistically improved in the group receiving additional vitamin C compared with the placebo group. The study confirms that the addition of oral vitamin C to therapy with atypical antipsychotic drugs may be beneficial in reducing clinical symptoms and restoring the balance between ROS and antioxidant defense in patients diagnosed with schizophrenia [10]. 

### 3.2. Impact of Vitamin C on Neurotransmission 

Vitamin C may affect neurotransmitter processes, including the synthesis and action of neurotransmitters such as dopamine, serotonin, and glutamate. Some studies suggest that disturbances in neurotransmitter systems may be associated with schizophrenia, so ascorbic acid may influence these systems and help regulate their function [11]. The aforementioned study by G. N. Dakhale et al. suggested that AA, as a potent antioxidant, may help mitigate the oxidative stress associated with dopamine activity, thereby potentially reducing the harmful effects linked to dopamine dysregulation in schizophrenia. As a result, high concentrations of ascorbic acid may potentially enhance the effects of drugs acting as dopamine receptor antagonists. The research results also suggest that ascorbic acid plays a crucial role in modulating the behavioral effects of atypical antipsychotic drugs. Simultaneous oral supplementation of vitamin C and atypical antipsychotic drugs may be beneficial in the therapy of schizophrenia [10]. 

### 3.3. Improvement of General Health 

Ascorbic acid is an important dietary component that helps maintain overall health and proper functioning of the body. There is ample evidence that a healthy diet can have a beneficial impact on mental health, including better functioning in schizophrenia [27]. Patients with schizophrenia have significantly lower fasting serum vitamin C concentrations and lower urinary excretion of vitamin C. In order to optimize vitamin C levels in patients with schizophrenia, AA supplementation was conducted for 4 weeks. After this period, serum vitamin C concentrations were similar in both groups, however, urinary excretion of vitamin C remained lower in patients with schizophrenia. These results reported by K. Suboticanec et al. suggest that there may be a mechanism linking the psychopathology of schizophrenia to abnormal AA metabolism [12]. Another study by K. Suboticanec et al. confirmed the hypothesis that patients with schizophrenia on the same basic diet as individuals in the control group required higher vitamin C concentrations than the suggested optimal requirement for AA in healthy individuals [13]. A study conducted by Supp et al. on an animal model of schizophrenia suggested that AA supplementation may be an effective method of preventing or alleviating some symptoms of schizophrenia induced by ketamine treatment, such as motor sensation, which may indicate its potential use as adjunctive therapy in schizophrenia treatment. In the experiment involving Wistar rats, animals received AA at different doses for 14 days, followed by administration of ketamine or saline. The group receiving AA supplementation showed less deterioration in motor and sensory functions compared with the control group receiving only saline. However, AA supplementation did not affect other parameters evaluated in the study [14]. Meanwhile, the case report presented by J.D. Kanofsky and R. Sandyk was about a patient with schizophrenia predominantly exhibiting negative symptoms such as blunted affect, social withdrawal, lack of motivation, impaired concentration, and cognitive dysfunction. The neuroleptic therapy (haloperidol 30 mg/day) initiated was not sufficiently effective. After introducing AA, the patient, who tended to leave the hospital and participated minimally in ward activities, became more motivated to participate in the educational program. Additionally, administration of vitamin E in combination with lorazepam further intensified these beneficial effects, leading to regular attendance in classes and a noticeable increase in the patient’s involvement in social life. It can be inferred that the synergistic interaction of these vitamins and basic treatment had a significant impact on improving his health condition [15]. Conclusions from a study conducted in 1987 by Linda Beauclair et al. suggested that AA could play an additional role in the treatment of schizophrenia. The study involved 13 patients with schizophrenia who were given 1 g/day of AA for the first 2 weeks, then 4 g/day for the next 2 weeks, and finally 8 g/day for the last 2 weeks of testing. The effects were evaluated using the Brief Psychiatric Rating Scale (BPRS) and the Clinical Global Impressions (CGI) scale. These findings may not be particularly significant or relevant due to the age of the study [16]. 

### 3.4. Interaction with Selected Neuroleptics and Potential Side Effects of AA Use

Several reports suggest that AA may be beneficial for patients treated with antipsychotic drugs such as clozapine. Some studies suggest that vitamin C may reduce some side effects of antipsychotic drugs [28]. Research has utilized the “open field” test to comprehensively examine various types of behaviors induced by ascorbic acid in combination with the typical antipsychotic drug haloperidol or atypical antipsychotic drugs such as clozapine, sulpiride, and remoxipride. Amineptine, an indirect dopamine agonist, was used in a model of dopaminergic activity. The results showed that amineptine dose-dependently increased locomotion and exploration. Ascorbic acid clearly inhibited mouse behavior and hyperactivity induced by amineptine. Combining each typical or atypical antipsychotic drug with amineptine induced a significant increase in walking and rearing compared with the effects observed with the antipsychotic drugs alone. However, combining antipsychotic drugs with ascorbic acid at a dose of 250 mg/kg led to a reduction in open field parameters compared with the control group. In summary, these data further confirm the in vivo impact of ascorbic acid on the dopaminergic system and demonstrate that the antidopaminergic effects of both typical and atypical antipsychotic drugs can be enhanced by simultaneous administration of ascorbic acid [17]. Similar conclusions were drawn by Deshpande C. et al., indicating that AA may have diverse effects on the dopaminergic system in the brain, depending on the dose. At low doses, AA exhibits dopaminergic agonist activity, while at high doses it acts as an antidopaminergic agent. Their study conducted on mice showed that AA enhanced the antipsychotic activity of both typical and atypical antipsychotic drugs. AA may interact with dopamine receptors and modulate the action of antipsychotic drugs such as haloperidol and clozapine [18]. G M Straw et al. conducted a study to investigate whether there were pharmacokinetic interactions between AA and haloperidol in a group of patients with schizophrenia. Eight men hospitalized with chronic schizophrenia, whose treatment was stabilized on a constant dose of haloperidol, were orally administered 4.5 grams of AA per day for 2 weeks in an open-label study. Psychiatric symptoms were monitored using the BPRS. Adding AA did not produce any changes in psychopathology in this group of patients [19]. Similarly, Ingole S et al. did not find statistically significant differences in parameters between patients receiving only olanzapine and patients receiving both olanzapine and vitamin C [20]. However, the hypothesis that vitamin C deficiency is a major risk factor for clozapine-induced agranulocytosis was put forward by Julia Ip et al. They assessed a guinea pig model with vitamin C deficiency. Clozapine is known to have a tendency to induce agranulocytosis, which limits its use. The mechanism of this phenomenon is partially related to the oxidation of clozapine via bone marrow cells to a nitrene ion. However, it was also found that vitamin C deficiency was probably not the main risk factor for clozapine-associated agranulocytosis [21]. On the other hand, Avril Pereira and Brian Dean conducted a cell culture study, specifically on bone marrow stromal cells, to assess the toxicity of clozapine and concluded that clozapine led to the formation of a toxic compound that could damage those cells, important for neutrophil development. Particularly noteworthy was the observation that AA was able to protect cells from clozapine toxicity, suggesting that vitamin C may played a role in preventing clozapine-induced bone marrow stromal damage [22]. Most commonly, vitamin C supplementation is not associated with serious side effects, especially if used in well-adjusted doses for a given patient, taking into account their laboratory tests. Supplementation for patients with schizophrenia is indicated due to the initial deficiency of this vitamin caused by the disease. Vitamin C is usually well tolerated by most individuals. In some cases, supplementation with high doses of vitamin C may cause gastrointestinal problems such as diarrhea, bloating, or abdominal pain. When exceeding the prescribed doses of vitamin C, kidney stones may form in predisposed individuals. High doses of vitamin C may affect the results of laboratory tests, which may lead to falsely elevated results in laboratory tests such as blood glucose tests or urine tests. Using large doses of vitamin C may interact with some medications, decreasing or increasing their effectiveness [23,24]. 

## 4. Results

Vitamin C may exhibit certain potential benefits as an adjunctive treatment in schizophrenia, although its exact impact is not fully understood. Some studies suggest that vitamin C may have antioxidant, anti-inflammatory, and neuroprotective effects, which may be associated with symptomatic improvement in schizophrenia. However, it should be noted that the available scientific literature contains a limited amount of research regarding the use of vitamin C in combination with antipsychotic medications in the treatment of schizophrenia.

### 4.1. Vitamin C as an Antioxidant

Research suggests that individuals diagnosed with schizophrenia may have elevated levels of oxidative stress, which can contribute to neuronal damage. Vitamin C, as a potent antioxidant, may help reduce this oxidative stress and protect nerve cells from damage. Additionally, vitamin C can inhibit the peroxidation of membrane phospholipids and act as a scavenger of free radicals. Significant reductions in ROS production have been observed in studies involving vitamin C. These results suggest that antipsychotic medications may lead to increased ROS production, which may be associated with their toxic effects; however, this depends on the dose and the cell systems and organs studied. Vitamin C has proven to be a significant cytoprotective factor against ROS.

### 4.2. Impact of Vitamin C on Neurotransmission

Vitamin C may influence neurotransmitter processes, including the synthesis and action of neurotransmitters such as dopamine, serotonin, and glutamate. Some studies suggest that disturbances in neurotransmitter systems may be associated with schizophrenia, so vitamin C may influence these systems and help regulate their function. Research findings indicate that vitamin C plays a significant role in modulating the behavioral effects of antipsychotic medications. Simultaneous oral supplementation of vitamin C and antipsychotic drugs may be beneficial in the therapy of schizophrenia.

### 4.3. Improvement of General Health

Vitamin C is an important dietary component that helps maintain overall health and proper functioning of the body. There is ample evidence that a healthy diet can have a beneficial impact on mental health, including better functioning in schizophrenia. Patients diagnosed with schizophrenia have significantly lower serum levels of vitamin C and lower urinary excretion of vitamin C. Supplementation with vitamin C may be an effective method of preventing or alleviating some symptoms of schizophrenia induced by ketamine treatment, such as motor sensation. Clinical cases also suggest that vitamin C supplementation may have a beneficial impact on the course of schizophrenia.

### 4.4. Interaction with Selected Neuroleptics and Potential Side Effects of Vitamin C Use

Research suggests that vitamin C may be beneficial for patients treated with antipsychotic drugs, such as clozapine. Findings from animal and cell studies suggest that vitamin C may protect against the toxicity of certain antipsychotic drugs, such as clozapine. However, it should be noted that supplementation with high doses of vitamin C may lead to adverse effects such as diarrhea, bloating, or abdominal pain.

## 5. Future Directions

The prospects for further research into the role of vitamin C in the treatment of schizophrenia are promising and encompass several key areas. Firstly, more extensive clinical studies are necessary, involving larger populations of patients with different types of schizophrenia, varying degrees of disease severity, and different ages. Such studies can help establish optimal dosages of vitamin C, treatment duration, and potential side effects. Additionally, long-term studies are needed to assess the effectiveness and safety of vitamin C supplementation over extended periods. This will help determine whether regular vitamin C supplementation can lead to long-term improvement in schizophrenia symptoms and whether any long-term side effects exist. Furthermore, studies on genetic differences can help identify patients with schizophrenia who may be more responsive to vitamin C supplementation. Variations in genes related to vitamin C metabolism may significantly affect the therapy’s effectiveness. 

## 6. Conclusions

In the era of evidence-based medicine, the results of studies could be used by psychiatrists to consider the potential benefits of vitamin C supplementation in the therapy of schizophrenia. Studies suggest that vitamin C may act as an antioxidant, anti-inflammatory, and neuroprotective agent, which may contribute to symptomatic improvement in schizophrenia. Additionally, interactions between vitamin C and antipsychotic drugs such as clozapine suggest potential reduction of some drug side effects and improvement in clinical assessment outcomes. These findings suggest that vitamin C supplementation may be a promising addition to the comprehensive therapy of schizophrenia, bringing potential benefits for both the mental health and overall health of patients. Although the conclusions drawn from the analysis of articles based on known mechanisms of action of vitamin C are quite general, reports on the possibility of ascorbic acid’s beneficial modulation of the action of antipsychotic drugs on schizophrenia symptoms are noteworthy, as they enhance the effectiveness of treatment rather than just reduce the intensity of potential side effects. 

## Figures and Tables

**Table 1:** Table showing the doses of vitamin c and clozapine reported in the publications.

Study	Details
Fakra E et al. [4]	Typical doses ranged from 300 to 900 mg daily, depending on individual tolerance and patient response to treatment.
Coward DM et al. [5]	Coward discussed the pharmacological effects of clozapine, noting that standard therapeutic doses ranged from 200 to 600 mg daily but may be adjusted based on the patient’s condition.
Heiser P et al. [9]	The aim of the examination was to demonstrate the effects of haloperidol, clozapine, and olanzapine in concentrations of 18, 90, and 180 μg/mL on the formation of ROS.
Dakhale GN et al. [10]	The dose of vitamin C was 500 mg/day.
Kocot J et al. [11]	The review described one dose—1000 mg/day.
Suboticanec K et al. [12]	Vitamin C status was determined in schizophrenic subjects using fasting plasma levels and the urinary dose response after an oral load of 1.0 g ascorbic acid.
Subotičanec K et al. [13]	Doses of 70 mg of AA for 4 weeks and 1 g AA for 4 weeks.
Supp AD et al. [14]	AA at 0.1, 1, and 10 mg/kg or saline for 14 days
Kanofsky JD et al. [15]	Vitamin C—6 g/day.
Beauclair L et al. [16]	The study used vitamin C as an adjunctive therapy in the treatment of schizophrenia. The doses used were 500 mg of vitamin C per day.
de Angelis L. [17]	Clozapine was used at a dose of 2.5 mg/kg intraperitoneally in combination with amineptine to observe behavioral changes.
Deshpande C et al. [18]	Vitamin C—2 mg/kg i.p.; Clozapine—1–2 mg/kg m.i.
Straw GM et al. [19]	The study assessed the effect of vitamin C (500 mg daily) on haloperidol concentrations in patients with schizophrenia, suggesting the potential benefit of vitamin C as an adjunctive therapy.
Shahu I et al. [20]	The study examined vitamin C supplementation (1000 mg daily) to prevent olanzapine-induced metabolic side effects in patients with schizophrenia.
Ip J, Wilson J et al. [21]	An animal study suggested that vitamin C deficiency may increase the risk of clozapine-induced agranulocytosis, but specific doses used in human studies were not reported.
Pereira A et al. [22]	The study considered the effect of clozapine oxidation by HOCl and the effect of system components on inhibition of HAS303 viability at various drug concentrations: (A) 42 μM clozapine, (B) 83 μM clozapine, and (C) 166 μM clozapine.
Myken AN et al. [23]	The study did not use doses of vitamin C but only examined its concentration in patients with mental illnesses.
Brown HE et al. [24]	The review described two doses—70 mg/day for four weeks in addition to antipsychotic treatment and 500 mg/day.
